# Weakly Hydrated Anion Exchangers Doped with Cu_2_O and Cu^0^ Particles—Thermogravimetric Studies

**DOI:** 10.3390/ma14040925

**Published:** 2021-02-15

**Authors:** Elżbieta Kociołek-Balawejder, Ewa Stanisławska, Igor Mucha

**Affiliations:** 1 Department of Industrial Chemistry, Wroclaw University of Economics and Business, Komandorska 118/120, 53-345 Wroclaw, Poland; elzbieta.kociolek-balawejder@ue.wroc.pl (E.K.-B.); ewa.stanislawska@ue.wroc.pl (E.S.); 2 Department of Analytical Chemistry, Wroclaw Medical University, Borowska 211 A, 50-556 Wroclaw, Poland

**Keywords:** hybrid ion exchanger, cuprous oxide, zero valent copper, thermal analysis, pyrolysis

## Abstract

Hybrid ion exchangers (HIXs) containing fine Cu_2_O and Cu^0^ particles were subjected to thermal analysis in order to determine their hygroscopic water content (with regard to their anomalously low porosity) and to determine the effect of the oxidation state of the copper atom in the deposit on the thermal properties of composite materials. Commercially available anion exchangers, Amberlite IRA 900Cl (macroreticular, M) and Amberlite IRA 402OH (gel-like, G), were used as supporting materials. M/Cu_2_O, G/Cu_2_O, M/Cu and G/Cu, containing 4.3–8.4 wt% Cu, were subjected to thermal analysis under respectively air and N_2_. TG/DTG curves revealed that dry M/Cu and G/Cu contained as little as 7.2% and 4.3% hygroscopic water, while M/Cu_2_O and G/Cu_2_O contained respectively 10.6% and 9.4% (Cu^0^ was a stronger water repellent than Cu_2_O). The oxidation state of the copper atom in the deposit was found to affect the amount of the forming char, and also Cu^0^ was found to contribute to the formation of more char than in the pyrolysis of the pure resin (the anion exchanger with no copper deposit). Under air the two kinds of particles transformed into CuO, while under N_2_ metallic copper and char (from the resin phase) made up the solid residue. This means that in the pyrolysis of the HIXs the inorganic phase participated in char formation and it also transformed itself (undergoing reduction when possible). The above findings provide a basis for in-depth research aimed at the innovative use of copper-containing HIXs and at obtaining usable composite materials with a designed (organic-inorganic) composition.

## 1. Introduction

Ion exchangers are used for purifying water, including in the treatment of water for conventional and nuclear power plants [1,2]. Many treatment and disposal strategies for reducing the environmental hazard arising from the generation of waste resins (some of which are radioactive waste) have been considered [3,4,5]. One of the ways of dealing with spent ion exchangers is their thermal decomposition, including incineration and pyrolysis, whereby the volume of the ion exchanger wastes is reduced and they acquire a more stable form for deposition in the environment [6,7,8,9,10,11,12]. Pyrolysis of ion-exchange resins yields porous carbon beads [13,14] usable for desulfurization of fuels [15], hydrogen storage [16], CO_2_ capture [17,18], sorption of heavy metal ions [19], benzene [20] and also in catalysis [21]. 

Ion exchangers containing dispersed inorganic fine particles, mostly of FeOOH [22,23] and also MnO_2_ [24] and ZrO_2_ [25,26] in their polymeric phase, are composite materials (called hybrid ion exchangers, HIXs) that offer properties and application opportunities not exhibited separately by the polymeric host materials or inorganic particles alone. Their polymeric-inorganic structure offers a synergy often aided by the Donnan membrane principle (in sorption processes the ions with charge similar to the functional groups are excluded from entering in the exchange phase while the ions with charge opposite to the functional groups tend to concentrate inside the exchanger phase [27]. HIXs show better properties, especially excellent sorptivity and selectivity in many reactions of environmental significance, than the parent resins [28]. They are used to remove such target contaminants as heavy metals, metalloids (especially arsenic), (in)organic ligands, fluoride, chlorophenols and pesticides, from water through sorption and redox processes [29]. 

A thermal analysis, comprising thermogravimetry (TG) and derivative thermogravimetry (DTG), needs to be carried out to identify the effect of the deposit on the thermal decomposition of the ion exchangers (the host materials). Recently we have undertaken in-depth studies into the thermal decomposition of HIXs. So far the studies have concentrated on anion exchangers containing particles of hydrated iron oxide (FeOOH) [30] and different cupric compounds (CuO, Cu(OH)_2_ and Cu_4_(OH)_6_SO_4_) in their porous matrix [31]. In comparison with the pure resins, the particles have been found to: (a) lower the incineration end temperature while raising the pyrolysis end temperature, (b) change the temperature ranges of the individual ion exchanger decomposition stages, (c) react with the char arising from organic matter (the anion exchanger) in the course of pyrolysis. Thanks to this latter property, stemming from the chemical structure of the inorganic phase (more precisely from its reducibility), it was possible to obtain more char than from the pyrolysis of the pure resin, and the char contained the chemically transformed (reduced when possible) inorganic phase [31]. Consequently, the pyrolysis of a HIX containing FeOOH yielded a carbonaceous material with Fe_3_O_4_ [30], the pyrolysis of a HIX containing Cu_4_(OH)_6_SO_4_ (brochantite) yielded a carbonaceous material with Cu_2_S and the pyrolysis of a HIX containing CuO yielded a carbonaceous material with metallic copper [31]. This means that by pyrolyzing HIXs one can obtain unconventional and potentially usable carbon char-inorganic particle composites with a designable chemical composition. Since HIXs may contain a considerable amount of inorganic phase (which can exceed 30 wt%) [32], causing pore narrowing and blockage and grain deformation and cracking during drying and so hindering gaseous phase access to the inside of the ion exchanger, we proposed to subject also freeze-dried samples retaining their open internal structure (which they had in the aqueous phase, i.e., in the natural operating conditions) to thermal analysis [30]. A similar phenomenon may occur in the case of inorganic composite materials when Cu^+^ ions penetrate inside the metal matrix, and the formation of Cu clusters causes a high strain of the host atoms [33]. 

In order to broaden and systematize our knowledge about the thermal properties of HIXs, including the effect of the oxidation state of the copper atom contained in the deposit on the properties, we investigated materials based on the same anion exchangers as previously, but containing fine particles of Cu_2_O [34] and Cu^0^ (zero valent copper, ZVC) [35]. The characteristic of the composite with Cu^0^, discovered in HIX for the first time, is the anomalous loss of the anion exchanger’s original porosity and strong volume contraction. Typically, macroreticular anion exchangers and the HIXs based on them have been known to exhibit (including when dried) porosity, which is an important parameter characterizing their performance (determined using the N_2_ adsorption-desorption method and mercury intrusion porosimetry) [32]. The materials investigated in this study differed from the most popular hydroscopic, porous materials containing hydrated oxides of polyvalent metals in the anion exchanger’s matrix.

The aim of this study was to examine the effect of dispersed Cu_2_O and Cu^0^ particles on the thermal decomposition of both macroreticular (M) and gel-like (G) anion exchangers in different atmospheres (under respectively air and N_2_) and to identify the solid products of the processes. More precisely, we wanted to answer the following questions: (a) What is the effect of the deposits on the hygroscopic water content in HIXs? (b) Do the deposits differ in their catalytic activity in decomposition of the anion exchanger? (c) Does the pyrolysis of the HIX with reducible Cu_2_O yield more char than the pyrolysis of the HIX with unreducible Cu^0^? and (d) Does the pyrolysis of the HIX with Cu^0^ yield a similar amount of char as the pyrolysis of the anion exchanger with no copper deposit?

The study is of both theoretical and practical importance as it shows that it is possible to sequentially (in stages) reduce the volume of spent HIXs in two different processes, presents a way of obtaining activated (doped with metallic copper) carbon sorbents through pyrolysis, and gives an insight into the catalytic properties of fine cuprous oxide and metallic copper particles deposited in the polymeric phase. Polymeric composites with copper-based additives (e.g., Cu, Cu_2_O, or CuO) are prospective materials for catalysis [36,37].

## 2. Materials and Methods

The polymer supports for Cu_2_O and Cu^0^ particles were Amberlite IRA 900Cl and Amberlite IRA 402OH, anion exchange resins produced by The Dow Chemical Company (Midland, MI, USA). First, starting resins were dried in a dryer chamber at 40 °C for 24 h. Four HIXs (Table 1) were obtained batchwise by treatment of the sample of anion exchanger (2.0 g) with the respective solution in mild conditions. The M/Cu_2_O and G/Cu_2_O were obtained by shaking at 20 °C for 24 h the corresponding anion exchanger in the form (filtered off under vacuum) with 40 cm^3^ of 0.5 mol∙dm^−3^ ascorbic acid in 2 mol∙dm^−3^ NaOH. The M/Cu and G/Cu were obtained by shaking at 50 °C for 3 h M/Cu_2_O and G/Cu_2_O with 80 cm^3^ of 1 mol∙dm^−3^ ascorbic acid. After each reaction the inorganic sediment (if it was present in the aqueous phase) was removed through decantation and the product (HIX), filtered off and washed with distilled water, was dried at 40 °C for 24 h. The content of Cu in HIXs was determined using inductively coupled plasma atomic emission spectroscopy and spectrophotometrically using cuprizone (bis(cyclohexanone)oxaldihydrazone) method [34,35].

### 2.1. Thermogravimetric Analysis

Thermogravimetric analysis was carried out using a TG 209 F1 Libra (Netzsch, Selb, Germany) thermobalance. For the materials tested in this work, approximately 12 mg of the samples were placed in ceramic crucibles (150 μL) and heated at a heating rate of 10 K/min. Thermogravimetric curves (TG) as well as the derivative curves (DTG) of the sample were obtained as an output of the experiment in the temperature range of 25–900 °C. Experiments were performed in a nitrogen atmosphere (30 mL/min) and in an oxidative atmosphere (protective gas—nitrogen 10 mL/min, and purge gas—air 30 mL/min). Netzsch Proteus 7.1.0 analysis software (Netzsch, Selb, Germany) was used to determine the mass change of the samples and DTG curves. Each measurement was performed three times to establish reproducibility. The temperatures and mass changes were given with an accuracy (standard deviation) of ± 0.5 K and 0.8% respectively.

### 2.2. Powder X-Ray Diffraction Analysis (XRPD)

The powder XRD measurements were performed on a Bruker D2 PHASER diffractometer (Bruker AXS, Karlsruhe, Germany) with a LynxEYe detector using Cu Kα1.2 radiation (1.5418 Å). Diffractograms were obtained in the Bragg-Brentano (θ/2θ) horizontal geometry between 5° and 70° (2θ) using a step scan mode with the step of 0.02° (2θ) and 0.2 s per step. All samples were studied in air at room temperature. The optics of the D2 PHASER diffractometer was a system of Soller slit module with 2.5°, a divergence slit with 0.6 mm and a nickel filter. The X-ray tube operated at 30 kV and 10 mA. The XRD patterns were processed using the software Diffrac.EVA v.3.2 (BRUKER AXS, Karlsruhe, Germany).

## 3. Result and Discussion

**Scheme 1:**
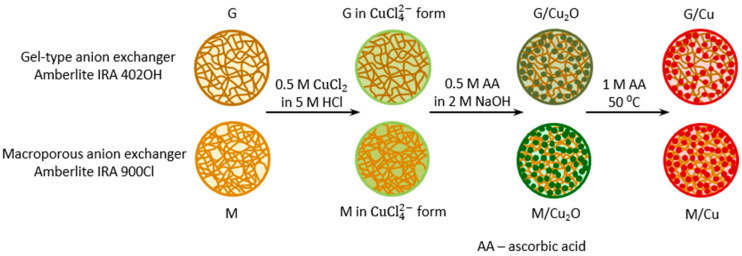
Formation of inorganic deposits in examined materials.

HIXs which have an identical polymeric skeleton but differ in the inorganic deposit identically distributed in this skeleton provide interesting material for study. Here the thermal decomposition of composites containing fine Cu_2_O and Cu^0^ particles in the matrix of respectively a macroreticular anion exchanger and a gel-like anion exchanger (Scheme 1) was compared. Since the Cu^0^ particles formed as a result of the reduction of the Cu_2_O particles previously deposited in the anion exchanger, one can assume that the two kinds of particles occupied similar sites in the matrix of the respective host materials.

Commercially available strongly basic anion exchangers Amberlite IRA 900Cl and Amberlite IRA 402OH, in the form of spherical beads, were used as supports for Cu_2_O and Cu^0^. The anion exchangers have a cross-linked polymeric matrix constituting a copolymer of styrene and divinylbenzene (macroreticular or gel-like) and quaternary ammonium functional groups –CH_2_N(CH_3_)_3_^+^. Their ion-exchange capacity (functional group content) is similar, amounting to about 3.0 meq∙g^−1^ (in the dry state). The anion exchangers were subjected to environmentally safe, operationally simple and inexpensive transformations (Scheme 1).

As the anion exchangers do not bond (but repel) Cu^2+^ cations (Cu_2_O and Cu^0^ precursors) from solutions of cupric salts, the key step in the synthesis was to transform their functional groups into the form and then to reduce the tetrachlorocuprate ions in such a way that the formed Cu_2_O particles would remain (not pass into the aqueous phase) in the ion exchanger’s matrix. This became possible owing to the use of a solution of ascorbic acid (AA) in NaOH excess [34]. In order to reduce Cu_2_O to Cu^0^ a solution of AA alone was used [35]. Table 1 shows four materials denoted as M/Cu_2_O, M/Cu, G/Cu_2_O and G/Cu, which were characterized in the previous studies. The difference in copper content between the HIXs containing Cu_2_O and the ones containing Cu^0^ was due to the different ionic form of the functional groups (hydroxylic vs. ascorbate, molar mass 17 vs. 175). Since anion exchangers show low thermal stability [38,39], all the materials investigated in this study were dried in mild conditions (at 40 °C for 24 h). The test results, grouped according to the structure of the host materials (M, G) and the thermal decomposition medium (air, N_2_), are presented in successive figures. Each of the figures shows numerical data characterizing the particular transformations, their temperature range and the temperature at which the maximum decomposition rate, mass change, end temperature and residual mass occurred.

**Figure 1:**
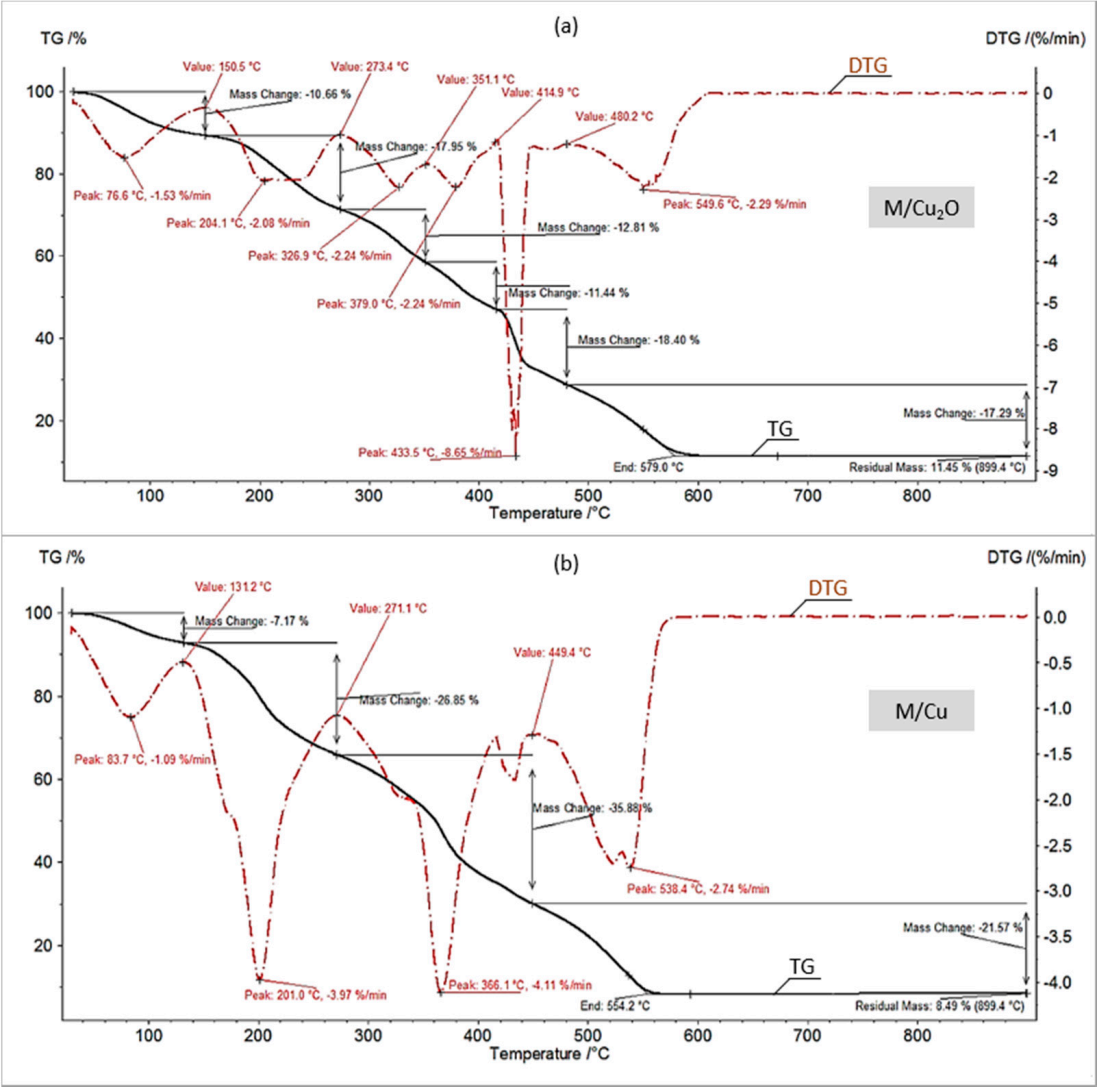
Thermogravimetry (TG)//derivative thermogravimetry (DTG) curves in air of (**a**) M/Cu_2_O, (**b**) M/Cu.

**Figure 2:**
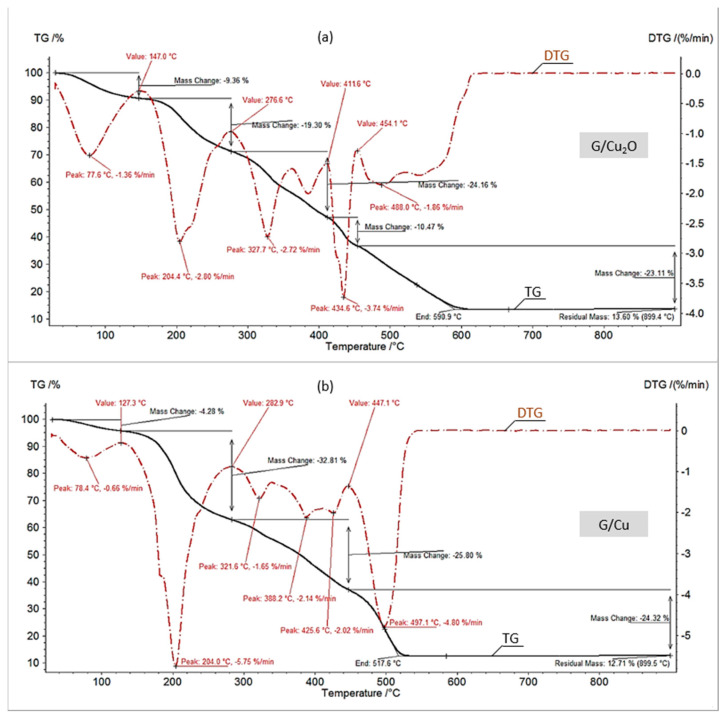
TG/DTG curves in air of (**a**) G/Cu_2_O, (**b**) G/Cu.

One of the most interesting aspects of this study was the assessment of the first step (dehydration) in the thermal decomposition of the investigated materials. In each of the four diagrams (Figure 1 and Figure 2, Table 2) the first change in the TG/DTG curves corresponded to the sample mass decrement due to water evaporation. It is apparent that the HIXs with Cu^0^ particles contained considerably less hydration bound water than the HIXs with Cu_2_O particles. This content for M/Cu (7.2%) and G/Cu (4.3%) was anomalously low as the pure resins (both anion exchangers) had about 15% hygroscopic water content [30,31]. Typically, in the parent resins (M, G) the functional ionogenic groups were surrounded by water molecules, which is referred to as solvation. This phenomenon is due to weak electrostatic interactions and results in the formation of a solvation envelope (a few water molecules thick) around polar functional groups [29,39,40]. After Cu^0^ was deposited in the anion exchanger’s matrix the configuration near the active groups changed. The shape of the TG/DTG curves indicated that this resulted in reduced solvation. In other words, the dry HIX with Cu^0^ retained less hygroscopic water than the dry anion exchanger (which means that the Cu^0^ deposit acted as a water-repellent).

Characteristically, the G/Cu samples contained less hydration bound water than the M/Cu samples. This can be explained by the fact that cross-linking reduces the retention of water in ion exchange resins. Gel-like anion exchangers contain less cross-linking agent (about 8% DVB) than macroreticular ion exchangers (about 15% DVB) and the fewer cross linkages an anion exchanger contains, the greater are its elasticity and swelling (this is why gel-like anion exchangers swell much more than macroreticular anion exchangers). Swelling and shrinking are reversible processes and are indeed accompanied by uptake/expulsion of water molecules [29,39]. Therefore one could suppose that in the presence of the water repelling agent the gel-like anion exchangers having a more elastic matrix shrank more than the macroreticular ones (the osmotic force and the elastic force act in opposite directions).

One might expect that the HIXs with Cu_2_O were poor in hygroscopic water too. The results confirmed this assumption only to a certain extent (Figure 1a and Figure 2a). The TG/DTG curves showed that the decrement in mass due to water evaporation for M/Cu_2_O (10.7%) and G/Cu_2_O (9.4%) was larger than in previously described experiments for HIXs with Cu^0^. This is in agreement with the FTIR analysis results for the examined materials, indicating that after the reduction of Cu_2_O to Cu^0^ the area of the broad peak indicative of adsorbed water at 3350 cm^−1^ decreased and almost vanished in the case of G/Cu [33]. One of the causes of the higher hygroscopicity of M/Cu_2_O and G/Cu_2_O in comparison with M/Cu and G/Cu could be the presence of functional groups in the OH^−^ form, which shows a very strong affinity for water [39]. It is worth mentioning than the HIXs with Cu_2_O particles contained clearly less hydration bound water (about 10%) than the HIXs with CuO particles (about 13%) [31].

In order to find out whether the fine particles precipitated in the polymeric skeleton of an anion exchanger affected its thermal decomposition (styrene-divinelbenzene copolymer with functional groups), the TG/DTG curves recorded at higher temperatures (after water evaporation) were compared. As Figure 1 and Figure 2 show, the thermal decomposition in air of the samples with Cu^0^ ended faster (at a lower temperature) than that of the samples with Cu_2_O, and both the composites lowered the polymeric phase decomposition end temperature in comparison with the parent resins (the anion exchangers with no deposit) [31]. Thus the decomposition of M, M/Cu_2_O and M/Cu ended at the temperatures of 594, 579 and 554 °C, while the decomposition of G, G/Cu_2_O and G/Cu terminated at the temperatures of 641, 591, and 518 °C, respectively. Interestingly, as regards the number of transformations (four stages) only the decomposition of M/Cu in air (Figure 1b) resembled the decomposition of M alone. As described in detail in our previous paper [30], the reference material, i.e., the macroreticular anion exchanger (M), decomposed in four stages which was due to water evaporation, functional group decomposition, benzene ring elimination from the styrene/divinylbenzene copolymer and the ultimate destruction (burning and incineration) of the polymeric matrix. In the case of M/Cu, the second stage of decomposition (the splitting off of the functional groups) proceeded at a relatively low temperature (in the range of 131–271 °C at the maximum decomposition rate of 4.2%/min at 201 °C, with a weight loss of 27%). As the functional groups of M/Cu were in the ascorbate form, it was concluded that the organic acid salt form (similarly as the OH^−^ form) accelerated (as opposed to the mineral acid salt form Cl^−^, SO_4_^2−^) the splitting off of the functional groups shifting this transformation by almost 100 °C towards higher temperatures [31]. This phenomenon could also have been the result of the catalytic (accelerating) action of Cu^0^ as the final two stages of the thermal decomposition of M/Cu also proceeded at a lower temperature in comparison with M alone.

**Figure 3:**
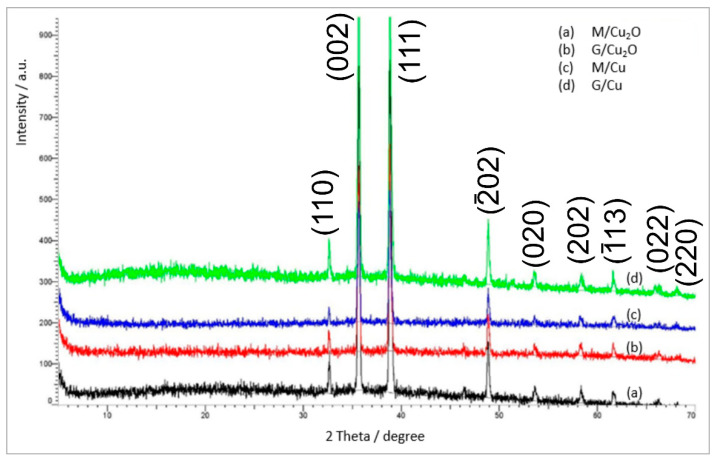
XRD patterns of the residues in air of (**a**) M/Cu_2_O, (**b**) G/Cu_2_O, (**c**) M/Cu, (**d**) G/Cu.

The TG/DTG curves under air for M/Cu_2_O, G/Cu_2_O and G/Cu (Figure 1a and Figure 2a,b) had a similar but unconventional shape, very different than for M/Cu (Figure 1b). At the temperature above 275 °C, after the functional groups have split off, one can see four transformations instead of two. The large number of small-area peaks indicates that the decomposition of the hydrocarbonaceous matrix was complicated due to the oxidation of Cu_2_O and Cu^0^ to CuO. An XRD analysis (Figure 3) showed CuO to be present in the products of the combustion of all the samples. The set of reflections at 2θ = 32.68, 35.73, 38.89, 48.91, 53.70, 58.29, 61.71, 66.04 and 68.25 was consistent with ICSD Card No. 16025.

**Figure 4:**
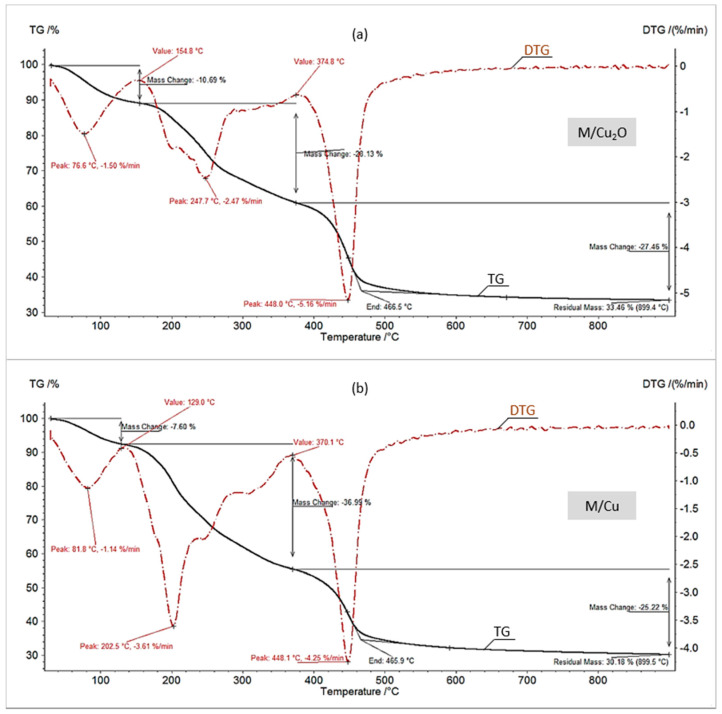
TG/DTG curves in N_2_ of (**a**) M/Cu_2_O, (**b**) M/Cu.

**Figure 5:**
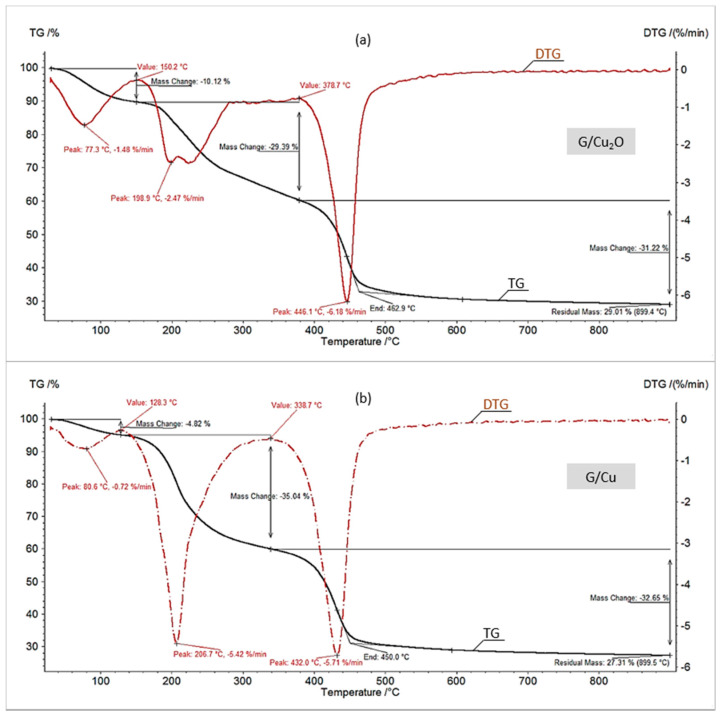
TG/DTG curves in N_2_ of (**a**) G/Cu_2_O, (**b**) G/Cu.

Then the TG/DTG curves recorded during pyrolysis (thermal decomposition under inert atmosphere) were analyzed. Figure 4 and Figure 5 show that the pyrolysis of all the samples proceeded similarly and typically for this process, i.e., in three stages: dehydration, the cleavage of the functional groups, and the decomposition of the polymer matrix [11,30,31]. The pyrolysis of all the samples would end at a temperature considerably lower than their combustion (about 450 °C vs. about 550 °C). Moreover, differently than in air, under nitrogen the presence of fine particles did not accelerate, but slightly slowed down the decomposition of the polymer matrix. Thus the effect of Cu^0^ and Cu_2_O on the pyrolysis of both the macroreticular anion exchanger and the gel-like one was to a large extent similar to the effect of CuO [31].

**Figure 6:**
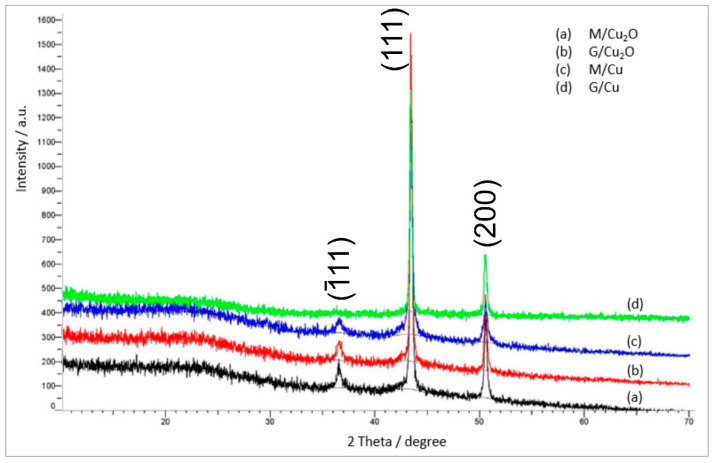
XRD patterns of the residues in N_2_ of (**a**) M/Cu_2_O, (**b**) G/Cu_2_O, (**c**) M/Cu, (**d**) G/Cu.

The most interesting aspects of the pyrolysis of the investigated materials were connected with the composition and amount of the solid residue (Table 2). The residue remaining after the pyrolysis of the HIXs (pyrolysate) had a much larger mass than the post-combustion residue (ash) since besides a mineral phase it contained an organic phase (char). More precisely, the pyrolysate contained char (a mixture of various high molecular weight organic compounds constituting a product of the decomposition of the polymer’s hydrocarbonaceous matrix in oxygen-free conditions) and inorganic matter (mainly deriving from the deposit). Our previous study showed that during pyrolysis the CuO particles contained in the HIXs had undergone reduction to Cu^0^ [31]. Therefore we expected a similar result in this study. Indeed, the XRD pattern (Figure 6) revealed that the inorganic load consisted mainly of metallic copper (peaks at 2θ = 43.44 and 50.57, ICSD Card No. 43493).

X-ray diffraction method provides a very simple possibility for estimating the crystallite size from the broadening of the XRD reflections via the Scherrer equation:

*D*_hkl_ = *Kλ*/(B_hkl_cos*θ*), (1)

where *D*_hkl_ is the crystallite size in a direction perpendicular to the reflection planes (hkl); *λ* is the X-ray wavelength (0.15418 nm); *K* is the Scherrer constant related to crystallite shape, normally taken as 0.94; B_hkl_ is the full-width at half-maximum in radians; and *θ* is the Bragg angle.

The most intensive diffraction peaks (111) of Cu and and (002) of CuO were analyzed to determine the particle size. The mean crystallites size of copper and copper oxide were determined as 48 nm and 58 nm correspondingly.

The residue remaining after the pyrolysis of M/Cu_2_O, M/Cu, G/Cu_2_O and G/Cu amounted to respectively about 31.2, 28.5, 26.8 and 24.3% of the original sample. As reported in our previous paper [31], the residue remaining after the pyrolysis of the reference materials (the anion exchangers with no copper deposit) amounted to respectively 11.2 (M) and 14.1% (G) of the original sample. A comparison of the above values provides interesting information about the essence of the pyrolysis of the HIXs. If one adds up the residue remaining after the pyrolysis of the reference material and the predicted (on the basis of the data presented in Table 1) metallic copper content in the product of the pyrolysis of a given material, one gets a result lower than the actual experimentally determined amount of residue. In the previous study we suggested a mechanism according to which an additional amount of char formed as a result of hydrogen expenditure for the reduction of cupric compounds (whereby there had been a hydrogen deficit in the reaction medium and the alkyl radicals, instead of generating volatile decomposition products, underwent condensation into products with larger non-volatile particles). In the present study the residue remaining after the pyrolysis of M/Cu_2_O and G/Cu_2_O (where the reduction of Cu_2_O to Cu^0^ had taken place) was larger than the residue remaining after the pyrolysis of M/Cu and G/Cu (where no reduction had taken place) but was clearly smaller than the residue remaining after the pyrolysis of M/CuO and G/CuO (where two-step reduction had taken place) [31].

**Scheme 2:**
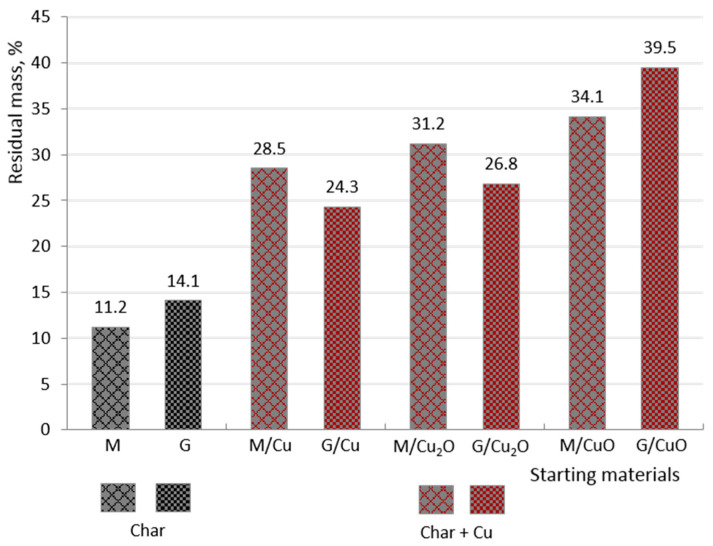
Solid residues in N_2_ of examined materials (M/Cu_2_O, M/Cu, G/Cu_2_O, G/Cu) in comparison to pure anion exchangers (M, G) and CuO doped anion exchangers (M/CuO, G/CuO) [31].

The following comparisons lead to interesting conclusions concerning the pyrolysis of the HIXs doped with copper containing deposit. The solid residues determined by us for the samples M/CuO, M/Cu_2_O, M/Cu and M form the series 34.1, 31.2, 28.5 and 11.2% respectively, while the series for G/CuO, G/Cu_2_O, G/Cu and G is 39.5, 26.8, 24.3 and 14.7% (Scheme 2).

The data indicate that the higher the oxidation state of the copper atoms present in the deposit was, the greater was the amount of the solid residue formed as a result of pyrolysis. In other words, since under N_2_ the transforming organic phase is capable of reducing the inorganic deposit, more of the latter condenses into products with large non-volatile particles than in the case of pure resin. The above observations do not fully explain the char formation mechanism as there was a significant difference in the amount of solid residue between M/Cu and M (and also between G/Cu and G), where no deposit reduction took place. One could suppose that because of the metallic copper’s catalytic properties (of vital importance in organic synthesis, especially for transformations of hydrocarbons [41]), under N_2_ considerably more char formed in the case of M/Cu than in the case of the same transformation of M. The higher amount of char in the pyrolysate of M/Cu and G/Cu than in the pyrolysate of the pure resin could also have been due to the functional groups in the ascorbate form (ascorbic acid is a carboxylic acid with a high molecular mass and it may have participated to some degree in char formation).

## 4. Conclusions

The thermal analysis of the HIXs with Cu^0^ particles showed that they contained anomalously little hygroscopic water, with amounts as low as about 4%. The HIXs with Cu_2_O particles contained about 10% of hygroscopic water, less than HIXs with CuO particles (13%), and considerably less than pure resin (15%). This means that Cu^0^ in the polymer matrix was a stronger water repellent than Cu_2_O and CuO.

Both Cu^0^ and Cu_2_O particles were shown to accelerate the decomposition of the anion exchanger in air, while slightly slowing down the decomposition of the anion exchanger in N_2_. Under air both the inorganic particles transformed into CuO, whereas under N_2_ the solid residue contained metallic copper. The oxidation state of the copper atom in the deposit was found to have an increasing effect on the amount of forming char, and metallic copper was found to contribute to the formation of a greater amount of char than in the case of the pyrolysis of the pure resin.

In brief: there are Cu-containing HIXs which when dried contain far less hygroscopic water than another similar composite materials. During the pyrolysis of HIXs with copper containing deposit (cupric compounds, cuprous compounds as well as metallic copper) the inorganic phase takes part in the formation of an additional amount of char and also transforms itself (reduces itself when possible). The two conclusions provide a basis for in-depth research aimed at the obtaining usable composites with a designed (organic-inorganic) composition and innovative use of this type materials (instead of synthetic carbon prepared on the basis of ion-exchange resin alone, by pyrolysis of HIX one can obtain metal-doped carbon possessing far more diverse properties).

## Figures and Tables

**Table 1:** Materials used for testing.

Properties	Symbol of HIX			
	M/Cu_2_O	M/Cu	G/Cu_2_O	G/Cu
Polymeric carrier	Amberlite IRA 900Cl		Amberlite IRA 402OH	
Physical form	Spherical beads			
Polymer matrix and functional groups	Styrene/divinylbenzene copolymer with quaternary ammonium groups			
Matrix structure	Macroreticular (M)		Gel-like (G)	
Inorganic deposit	Cu_2_O	Cu^0^	Cu_2_O	Cu^0^
Cu content in HIX, wt%	8.40	6.86	6.54	4.30
Form of functional groups	$OH^{-}$	ascorbate	$OH^{-}$	ascorbate

**Table 2:** Statement of thermal decomposition results.

Symbol of HIX	Water Mass Loss, %	Peak Temperature of Polymer Degradation, °C	End Temperature, °C	Residual Mass, %
Decomposition in air				
M/Cu_2_O	10.66	204.1, 326.9, 433.5, 549.6	579.0	11.45
M/Cu	7.17	201.0, 366.1, 538.4	554.2	8.49
G/Cu_2_O	9.36	204.4, 327.7,434.6, 4.88	590.9	13.60
G/Cu	4.28	204.0, 321.6, 425.6, 497.1	517.6	12.71
Decomposition in N_2_				
M/Cu_2_O	13.61	245.3, 448.4	467.4	31.21
M/Cu	8.54	201.6, 448.9	469.4	28.51
G/Cu_2_O	13.22	196.5, 446.6	466.0	26.83
G/Cu	6.76	207.4, 435.8	456.4	24.31

## Data Availability

The data presented in this study are available on request from the corresponding author.
